# Seven new species of *Trigonopterus* Fauvel (Coleoptera, Curculionidae) from the Tanimbar Archipelago

**DOI:** 10.3897/zookeys.888.38642

**Published:** 2019-11-11

**Authors:** Raden Pramesa Narakusumo, Michael Balke, Alexander Riedel

**Affiliations:** 1 State Museum of Natural History Karlsruhe, Erbprinzenstr. 13, D-76133 Karlsruhe, Germany; 2 Museum Zoologicum Bogoriense, Research Center for Biology, Indonesian Institute of Sciences (LIPI), Gd. Widyasatwaloka, Jl. Raya Jakarta-Bogor km 46, Cibinong 16911, Indonesia; 3 SNSB-Zoological State Collection (ZSM), Münchhausenstr. 21, D-81247 Munich, Germany

**Keywords:** Coleoptera, conservation, *cox1*, Cryptorhynchinae, DNA barcoding, endemism, hyperdiverse, integrative taxonomy, Moluccas, morphology, Southeast Asia, Tanimbar, turbo-taxonomy, Wallacea, weevils.

## Abstract

Based on recent fieldwork, the hyperdiverse weevil genus *Trigonopterus* Fauvel is recorded for the first time from the Indonesian Tanimbar Archipelago, halfway between Australia and Western New Guinea. All seven species discovered on Tanimbar are new to science, and described here: *Trigonopterus atuf***sp. nov.**, *T. kumbang***sp. nov.**, *T. laratensis***sp. nov.**, *T. porg***sp. nov.**, *T. selaruensis***sp. nov.**, *T. tanimbarensis***sp. nov.**, and *T. triradiatus***sp. nov.** The new species are authored by the taxonomists-in-charge, Raden Pramesa Narakusumo and Alexander Riedel. This fauna appears discordant and established by relatively recent dispersal from New Guinea and other Moluccan islands.

## Introduction

*Trigonopterus* Fauvel is a genus of hidden snout weevils (Cryptorhynchinae) (Alonso-Zarazaga and Lyal 1999; Riedel et al. 2016). These beetles are flightless, yet the 444 species currently known cover a large geographic area, across the Indo-Australian Archipelago and into Oceania (Riedel et al. 2010, 2014; Riedel and Tänzler 2016; van Dam et al. 2016). Hundreds of additional species await discovery (Riedel et al. 2013b, 2014; Riedel and Tänzler 2016; Riedel and Narakusumo 2019; https://species-id.net/wiki/Trigonopterus; unpublished data). New Guinea is the center of *Trigonopterus* diversity (Tänzler et al. 2012, 2016, 2017).

The relatively rich *Trigonopterus* faunas of Sulawesi and Sundaland originated by dispersal from New Guinea and subsequent diversification (Tänzler et al. 2016). The fauna of the Moluccan Islands may have served as stepping stones, yet little is known about these islands. There is only one described species from Seram Island, i.e., *Trigonopterus ellipticus* (Pascoe) (Riedel 2011), though several undescribed species from Halmahera and Ternate Islands were included in the phylogeny of the genus published by Tänzler et al. (2016).

Here, we present the results of a recent survey of the Tanimbar Archipelago, or simply Tanimbar. Tanimbar is a cluster of islands located approximately halfway between Australia in the south (Darwin area, ca. 320 km distant) and Western New Guinea in the north (ca. 340 km). The island of Timor is ca. 380 km to the west, and the Kai and Aru Islands lie ca. 150 km and 240 km, respectively, to the northeast. The Tanimbar Islands are all low, i.e., below an elevation of 300 meters. The climate is relatively seasonal, and forest cover comprises of seasonal evergreen forest, dry deciduous forest and moist deciduous forest (Laumonier and Nasi 2018). Geologically, Tanimbar belongs to the outer non-volcanic Banda arc formed in the Quaternary (Hall 1998, 2002). Parts of the islands are covered with early Pleistocene marine deposits and quaternary reefs occur up to 200 m in altitude (De Smet et al. 1989; Charlton et al. 1991), indicating a very recent origin of ca. 1 Ma. During the Pleistocene, the Tanimbar Islands remained insular as they are not connected to the Sahul shelf (Voris 2000). Thus, Tanimbar has been used as a geological calibration point for a phylogenetic analysis of passerine birds (Jønsson et al. 2010).

Here we describe seven new species of *Trigonopterus* from Yamdena, Larat, and Selaru islands, the three biggest islands of the Tanimbar Archipelago. We follow the “fast-track” taxonomy approach that combines molecular and morphological systematics (Riedel et al. 2013a, b), including data release on open access websites, i.e., species-ID (https://species-id.net/wiki/Trigonopterus) and wikispecies (https://species.wikimedia.org/wiki/Trigonopterus).

## Materials and methods

This study is based on 222 specimens of *Trigonopterus* collected on two field trips to the Tanimbar Islands by the first author. Specimens were collected by beating foliage in primary forest. Holotypes were selected from 44 DNA sequenced specimens. DNA was extracted nondestructively as described by Riedel et al. (2010), with proteinase K lysis so that the genitalia of most specimens did not require extra maceration after DNA-extraction and could be directly stained with an alcoholic Chlorazol Black solution and stored in glycerol in microvials attached to the pin of the specimens. Genitalia of collection specimens or specimens whose abdominal muscle tissue was not sufficiently digested after DNA extraction were macerated in a 10% KOH solution and rinsed in diluted acetic acid before staining. Illustrations of habitus and genitalia were prepared from holotypes. Finally, type series were supplemented with specimens stored in ethanol and older material from the dry collection. Type depositories are cited using the following codens:

**MZB**LIPI Research Center of Biology, Division of Zoology, Museum Zoologicum Bogoriense, Widyasatwaloka, Cibinong, Indonesia;

**SMNK**Staatliches Museum für Naturkunde, Karlsruhe, Germany;

**ZSM** Zoologische Staatssammlung München, Germany.

The methods applied for DNA sequencing and sequence analysis are described by Riedel et al. (2010) and Tänzler et al. (2012). Morphological descriptions are limited to major diagnostic characters as outlined by Riedel et al. (2013a, b). Negative character states (i.e., the absence of a character) are only mentioned explicitly where it appears appropriate. In groups comprising hundreds of species enumerating the absence of rare character states leads to inflated descriptions that distract the reader from the important information, i.e., the diagnostic characters present in a given species.

The closest relatives of Tanimbar species were identified by creating an alignment of 1.154 *cox1* sequences representing ca. 1000 species and generating a maximum likelihood reconstruction using the program IQTREE (Nguyen et al. 2015, Trifinopoulos et al. 2016). Morphological terminology follows Beutel and Leschen (2005) and Leschen et al. (2009), i.e., the terms “mesoventrite” / “metaventrite” are used instead of “mesosternite” / “metasternite” and “mesanepisternum” / “metanepisternum” instead of “mesepisternum” / “metepisternum”; “penis” is used instead of “aedeagus” as the tegmen is usually without useful characters in *Trigonopterus* and therefore omitted from species descriptions. Specimens were examined with a Leica MZ16 dissecting microscope and a fluorescent desk lamp for illumination. Measurements were taken with the help of an ocular grid. The length of the body was measured in dorsal aspect from the elytral apex to the front of the pronotum. Legs were described in an idealized laterally extended position; there is a dorsal / ventral and an anterior / posterior surface. Habitus illustrations were compiled using a DFC495 camera with L.A.S. 4.8.0 software adapted to a Z6 APO (all from Leica Microsystems, Heerbrugg, Switzerland). Photographic illustrations of genitalia were made using a DFC450 camera with L.A.S. 4.8.0 software adapted to an Axio Imager M2 microscope (Carl Zeiss Microscopy), with 5×, respectively 10× A-Plan lenses; resulting image stacks were compiled using the Helicon Focus 6.7.1 Pro software (Helicon Soft Ltd). For photography genitalia were temporarily embedded in glycerol gelatin as described by Riedel (2005), with their longitudinal axis somewhat lifted caudally, to adequately illustrate structures of the curved down apex. All photographs were enhanced using the programs Adobe Photoshop CS2 and CS6. However, care was taken not to obscure or alter any features of the specimens illustrated. Sequence data were submitted to GenBank of NCBI (National Center for Biotechnology Information) and the accession numbers are provided under each species, e.g., as “(EMBL # MN322570)”.

## Taxonomy

### Trigonopterus

Taxon classificationAnimaliaColeopteraCurculionidae

Fauvel, 1862

45051FAD-8240-59F8-A473-0F927DE4CF0E

#### Type species, by monotypy.

*Trigonopterus insignis* Fauvel, 1862.

#### Diagnosis.

Fully apterous genus of Cryptorhynchinae s. s. Length 1.5–6.0 mm. Rostrum in repose not reaching middle of mesocoxa. Scutellar shield completely absent externally. Mesothoracic receptacle deep, posteriorly closed. Metanepisternum completely absent externally. Elytra with nine striae (sometimes superficially effaced). Tarsal claws minute. Usually body largely unclothed, without dense vestiture. For additional information, see http://species-id.net/wiki/Trigonopterus.

##### Descriptions of species

### Trigonopterus atuf

Taxon classificationAnimaliaColeopteraCurculionidae

Narakusumo & Riedel sp. nov.

4443A0CA-D177-5F1C-ACCE-5B797464D181

http://zoobank.org/1367A851-B3E3-4A64-9463-73A828646D9B

#### Diagnostic description.

**Figure 1.**
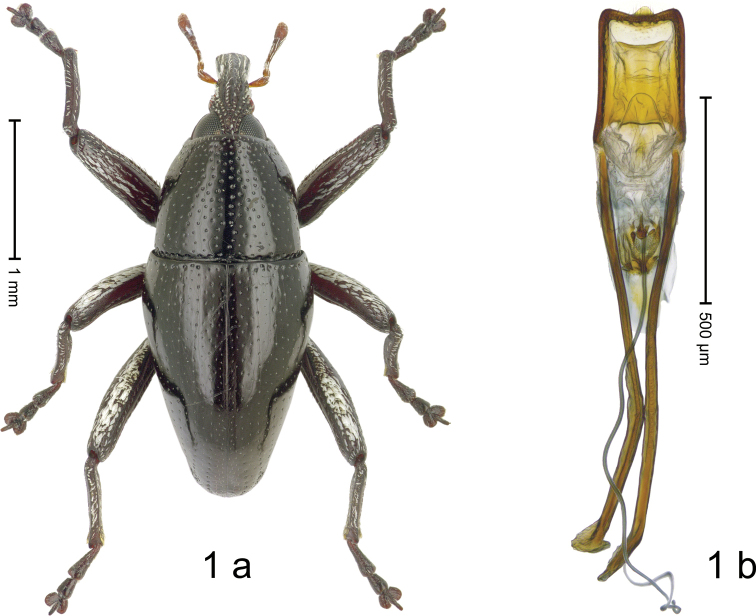
*Trigonopterus atuf* Narakusumo & Riedel, sp. nov., holotype **a** habitus **b** penis.

***Holotype*. Male** (Fig. 1a). Length 2.65 mm. Color of antennae and legs ferruginous, remainder black. Body elongate subovate; profile dorsally convex; in dorsal aspect and in profile with weak constriction between pronotum and elytron. Eyes dorsally approximate. Rostrum with median carina and pair of submedian ridges, intervening furrows with suberect scales. Pronotum subglabrous, punctate. Elytra subglabrous, striae marked by rows of minute punctures and fine hairlines; along base towards humerus bordered by transverse row of deeper punctures; intervals subglabrous, with sparse minute punctures; subapically along ventral margin with row of white scales. Femora with distinct anteroventral ridge, edentate. Mesofemur and metafemur dorsally densely squamose with white scales. Metafemur with smooth dorsoposterior edge; subapically without stridulatory patch. Procoxa with patch of erect white scales. Abdominal ventrites 1–2 concave, medially subglabrous, laterally with sparse white scales; ventrite 5 with shallow impression, punctate, with sparse suberect scales. Penis (Fig. 1b) with sides subparallel, apex subtruncate, with sparse setae, medially with angulate extension; apodemes 3.0× as long as body of penis; transfer apparatus complex; ductus ejaculatorius without bulbus. **Intraspecific variation.** Length 2.30–2.78 mm. Female rostrum subglabrous, punctate-rugose; in basal quarter with suberect scales. Female abdominal ventrite 5 flat.

#### Material examined.

***Holotype*** (MZB): MZB0014 (GenBank # MN322570), Indonesia, Maluku, Tanimbar, Yamdena Is, Lorulun, 07°48.788'S, 131°22.443'E to 07°48.137'S, 131°21.873'E, 140 m, beaten, 2-V-2017. ***Paratypes*** (MZB, SMNK): Indonesia, Maluku, Tanimbar: 13 exx, MZB0012 (EMBL # MN322578) MZB0013 (GenBank # MN322580) MZB0015 (GenBank # MN322569) same data as holotype; 3 exx, MZB0017 (GenBank # MN322568), MZB0018 (GenBank # MN322567), MZB0024 (GenBank # MN322579), Selaru Is, Bangruti, 08°07.253'S, 131°02.947'E, 35 m, beaten, 22-IV-2018; 5 exx, MZB0031 (GenBank # MN322577), MZB0032 (GenBank # MN322576), MZB0040 (GenBank # MN322571), Yamdena Is, Lorulun, Jungle Camp, 07°46.46'S, 131°20.482'E, 110 m, beaten, 19-IV-2018; 1 ex, MZB0034 (GenBank # MN322574), Yamdena Is, Lorulun, Jungle track, 07°47.396'S, 131°20.849'E, 120 m, beaten, 19–20-IV-2018; 47 exx MZB0038 (GenBank # MN322573), MZB0039 (GenBank # MN322572), Yamdena Is, Lorulun, Jungle camp, 07°46.46'S, 131°20.482'E, 110 m, beaten, 24-IV-2018; 2 exx, MZB0033 (GenBank # MN322575), Yamdena Is, Lorulun, Jungle camp, 07°46.46'S, 131°20.482'E, 110 m, beaten, 27-IV-2018.

#### Distribution.

Maluku Prov., Tanimbar (Yamdena Is, Selaru Is). Elevation: 35–120 m.

#### Biology.

On foliage in lowland forest.

#### Etymology.

The epithet is a noun in apposition. Atuf is a mythical warrior from the folklore of the Tanimbar people who defeated the sun.

#### Notes.

This species is closely related to *Trigonopterus* species 773 from New Guinea, which differs by having a more distinct punctation and 15.1% p-distance of its *cox1* sequence.

### Trigonopterus kumbang

Taxon classificationAnimaliaColeopteraCurculionidae

Narakusumo & Riedel sp. nov.

67EF26F6-84DE-5B1A-B325-3694CC35C3F1

http://zoobank.org/59F9DD00-C782-49EC-B32C-E53DD8F5ABB9

#### Diagnostic description.

**Figure 2.**
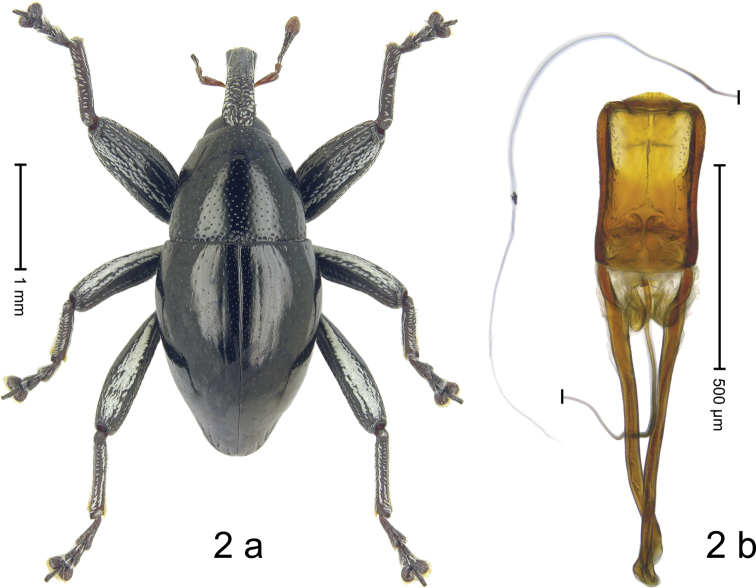
*Trigonopterus kumbang* Narakusumo & Riedel, sp. nov., holotype **a** habitus **b** penis.

***Holotype*. Male** (Fig. 2a). Length 3.12 mm. Color of antennae ferruginous, remainder black. Body subovate; in dorsal aspect and in profile with weak constriction between pronotum and elytron. Eyes dorsally approximate. Rostrum in basal 1/2 dorsally weakly swollen, with median ridge, coarsely punctate, with dense white scales; in apical half flat, subglabrous, sparsely punctate. Pronotum subglabrous, sparsely punctate with small punctures, somewhat denser and larger along basal margin. Elytra subglabrous, with sparse minute punctures, laterally and subapically striae marked by sparse rows of small punctures; stria 8 along humerus with short row of seven deeper punctures. Femora with distinct anteroventral ridge, near middle with denticle. Mesofemur and metafemur dorsally densely squamose with white scales. Metafemur with smooth dorsoposterior edge; subapically without stridulatory patch. Mesocoxa with densely squamose patch. Abdominal ventrites 1–2 concave, subglabrous, laterally with sparse white scales; ventrite 5 flat, subglabrous, microreticulate, with minute punctures, laterally with coarse punctures and sparse white scales. Penis (Fig. 2b) with sides subparallel, apically subangulate. Transfer apparatus simple, dentiform. Apodemes 2.6× as along as body of penis; ductus ejaculatorius with indistinct bulbus. **Intraspecific variation.** Length 3.12–3.53 mm. Female rostrum in apical 2/3 dorsally flat, subglabrous, with minute punctures; in basal 1/3 dorsally swollen, coarsely punctate. Female abdominal ventrites 1–2 flat.

#### Material examined.

***Holotype*** (MZB): MZB0002 (GenBank # MN322581), Indonesia, Maluku, Tanimbar, Yamdena Is, Lorulun, 07°48.788'S, 131°22.443'E to 07°48.82'S, 131°21.524'E, 140 m, beaten, 29-IV-2017. ***Paratypes*** (MZB, SMNK): Indonesia, Maluku, Tanimbar: 7 exx, MZB0001 (GenBank # MN322586), MZB0003 (GenBank # MN322585) same data as holotype; 1 ex, MZB0026 (GenBank # MN322582), Yamdena Is, Lorulun, Jungle Camp, 07°46.46'S, 131°20.482'E, 112m, beaten, 24-IV-2018; 3 exx, MZB0042 (GenBank # MN322584), Larat Is, Nature Reserve, 07°08.747'S, 131°49.092'E, 90 m, beaten, 25–26-IV-2018; 1 ex, MZB0019 (GenBank # MN322583), Larat Is, Nature reserve, 07°08.747'S, 131°49.092'E, 90 m, beaten, 26-IV-2018; 5 exx, Yamdena Is, Lorulun, 07°48.473'S, 131°22.266'E to 07°48.137'S, 131°21.873'E, 140 m, beaten, 02-V-2017.

#### Distribution.

Maluku Prov., Tanimbar (Yamdena Is, Larat Is). Elevation 90–140 m.

#### Biology.

On foliage in lowland forest.

#### Etymology.

This epithet is the Indonesian word for beetle and a noun in apposition.

#### Note.

This species is closely related to the undescribed *Trigonopterus* species 929 (*T. nasutus*-group) from the D´Entrecasteaux Islands from which it differs by its smaller body size, a subglabrous side of the pronotum, and a 12.3% p-distance of its *cox1* sequence.

### Trigonopterus laratensis

Taxon classificationAnimaliaColeopteraCurculionidae

Narakusumo & Riedel sp. nov.

75B5741D-3538-55D0-AFAC-84D8F118A6CB

http://zoobank.org/C27ED24F-2114-478D-AFBA-3256ECE4BD4B

#### Diagnostic description.

**Figure 3.**
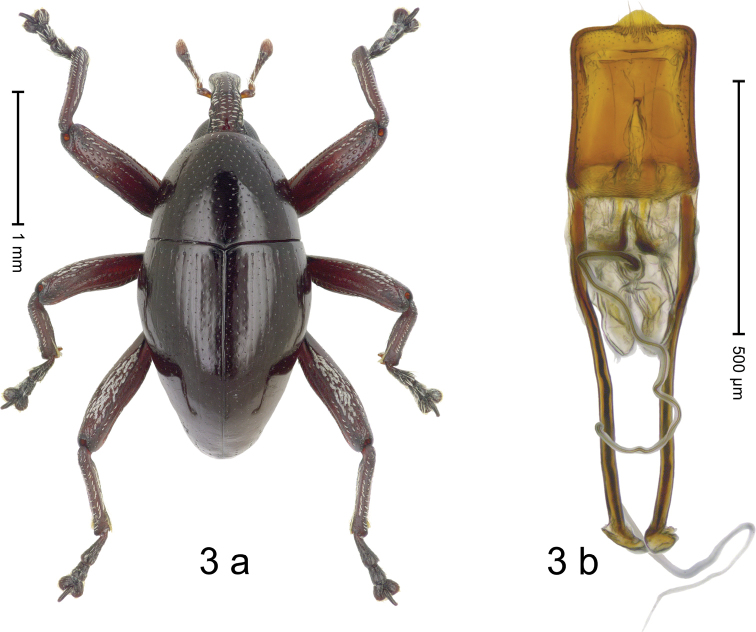
*Trigonopterus laratensis* Narakusumo & Riedel, sp. nov., holotype **a** habitus **b** penis.

***Holotype*: Male** (Fig. 3a). Length 2.53 mm. Color of head, femora and tibiae ferruginous; remainder black. Body subovate; in dorsal aspect almost without constriction between pronotum and elytron; profile dorsally convex. Rostrum in basal 1/2 with median costa and submedian ridges, intervening furrows with sparse, suberect scales. Pronotum subglabrous, sparsely punctate with small to minute punctures; anterolaterally with coarse punctures. Elytra subglabrous, punctate with minute punctures; striae marked by faint hairlines. Femora subglabrous weakly microreticulate, with minute punctures; with anteroventral ridge distinct, simple. Meso- and metafemur dorsally squamose with white scales. Mesotibia basally rounded; subapically with uncus and larger premucro. Metafemur subapically simple, without stridulatory patch; with uncus, without premucro. Abdominal ventrites 1–2 medially concave, subglabrous, microreticulate; ventrite 2 posteriorly projecting and forming edge; ventrite 5 almost flat, weakly concave, microreticulate. Penis (Fig. 3b) with side subparallel; apex symmetrical, with median triangular extension; transfer apparatus dentiform, apically bordered by pair of L-shaped sclerites; apodemes 3.0× as long as body of penis; ductus ejaculatorius with indistinct bulbus. **Intraspecific variation.** Length 2.48–2.53 mm. Female rostrum dorsally subglabrous, punctate; in basal 1/3 with median and submedian ridges. Female mesotibia subapically with uncus and minute premucro. Female abdominal ventrites 1–2 flat, with sparse punctures; ventrite 5 flat, subglabrous.

#### Material examined.

Holotype (MZB): MZB0022 (GenBank # MN322587), Indonesia, Maluku, Tanimbar, Larat Is, Nature reserve, 07°08.747'S, 131°49.092'E, 85 m, beaten, 25–26-IV-2018. Paratype (SMNK): Indonesia, Maluku, Tanimbar: 1 ex, MZB0027 (GenBank # MN322588), Yamdena Is, Lorulun, Jungle Camp, 07°46.46'S, 131°20.482'E, 110 m, beaten, 24-IV-2018.

#### Distribution.

Maluku Prov., Tanimbar (Yamdena Is, Larat Is). Elevation: 85–110 m.

#### Biology.

On foliage in lowland forest.

#### Etymology.

This epithet is based on the type locality Larat Island.

#### Notes.

This species belongs to the *T. politus* group. It is most closely related to a clade comprising *T. allotopus* Riedel, *T. pseudallotopus* Riedel, and some undescribed species from New Guinea, but it has no close relationship to the clade of Australian species.

### Trigonopterus porg

Taxon classificationAnimaliaColeopteraCurculionidae

Narakusumo & Riedel sp. nov.

566CDAF7-62F5-5142-BEA8-F4979C750F66

http://zoobank.org/96024735-BEC6-4650-BFB4-075551983D81

#### Diagnostic description.

**Figure 4.**
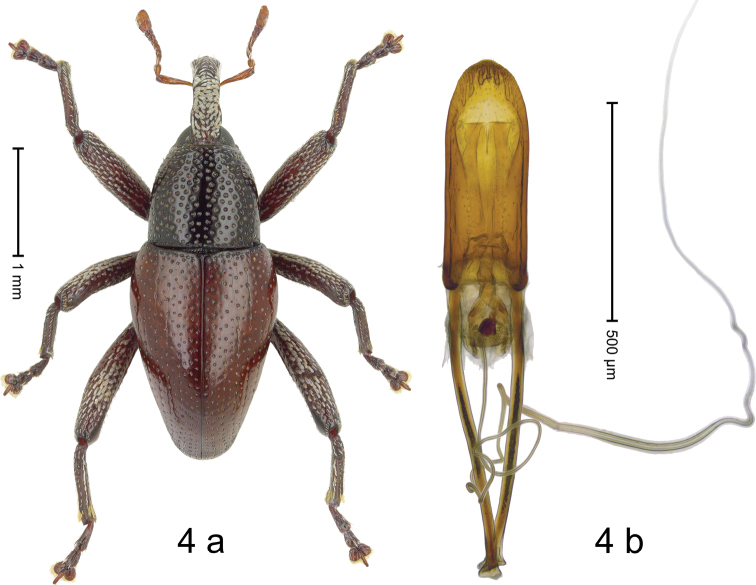
*Trigonopterus porg* Narakusumo & Riedel, sp. nov., holotype **a** habitus **b** penis.

***Holotype*. Male** (Fig. 4a). Length 2.98 mm. Color of antennae, legs and elytra ferruginous, remainder black. Body elongate; in dorsal aspect and in profile with moderate constriction between pronotum and elytron. Rostrum in basal 2/3 with median carina, dorsal surface clothed with white scales; in apical 1/3 subglabrous, punctate-rugose, with suberect setae. Pronotum densely punctate, with large punctures, with subglabrous midline; anterolaterally with white scales. Elytra subglabrous with irregular small punctures; few striae faintly marked by hairlines. Femora with distinct anteroventral ridge, ending near middle with small tooth. Meso- and metafemur dorsally with silvery scales. Metafemur subapically with stridulatory patch. Abdominal ventrites 1–2 concave, subglabrous, posteriorly sparsely punctate, with sparse scales; ventrite 5 weakly concave with sparse setae. Penis (Fig. 4b) with sides subparallel, in apical 1/3 converging to subangulate apex; transfer apparatus denticulate; apodemes 1.6× as long as body of penis; ductus ejaculatorius with distinct bulbus. **Intraspecific variation.** Length 2.75–2.98 mm. Female rostrum with subglabrous median costa, laterally punctate-rugose, with short setae. Female pronotum anterolaterally with scales transparent-yellowish. Female abdominal ventrites 1–2 concave, subglabrous with short setae. Female ventrite 5 flat.

#### Material examined.

***Holotype*** (MZB): MZB0043 (GenBank # MN322591), Indonesia, Maluku, Tanimbar, Larat Is, Nature reserve, 07°08.747'S, 131°49.092'E, 85 m, beaten, 25–26-IV-2018. ***Paratypes*** (MZB, SMNK): Indonesia, Maluku, Tanimbar: 4 exx, MZB0016 (GenBank # MN322590), MZB0044 (GenBank # MN322589) same data as holotype.

#### Distribution.

Maluku Prov., Tanimbar (Larat Is). Elevation ca. 85 m.

#### Biology.

On foliage in lowland forest.

#### Etymology.

This epithet is a noun in apposition based on the fictional penguin-like character Porg in the Star Wars movies. This species inhabiting a remote island has the same color combination of black, orange and white.

#### Notes.

This species is closely related to the undescribed species 437 from Kai Kecil Island from which it differs by the elytral color and a 13.6% p-distance of its *cox1* sequence.

### Trigonopterus selaruensis

Taxon classificationAnimaliaColeopteraCurculionidae

Narakusumo & Riedel sp. nov.

D28A5109-25FA-5357-AD74-E8DC4AF95722

http://zoobank.org/A9B7F81C-432D-40A7-B99C-688C6F4BEA44

#### Diagnostic description.

**Figure 5.**
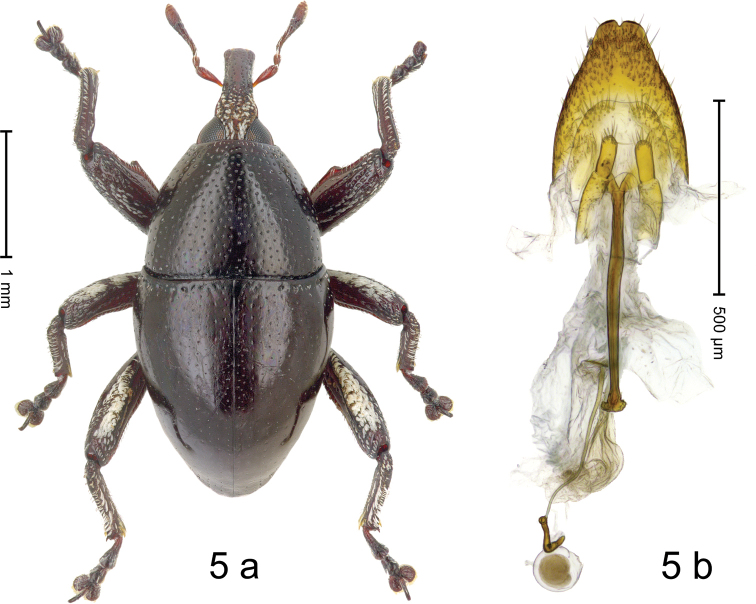
*Trigonopterus selaruensis* Narakusumo & Riedel, sp. nov., holotype **a** habitus **b** female genitalia.

**Figure 6.**
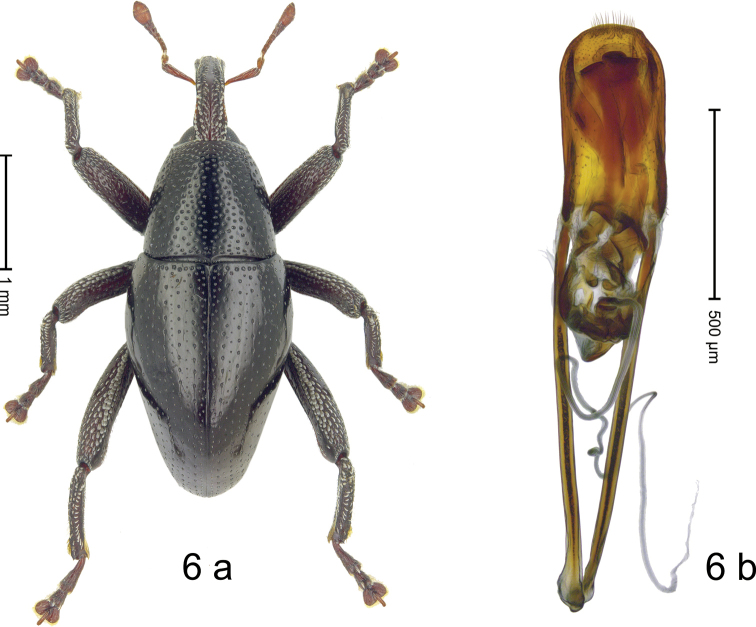
*Trigonopterus tanimbarensis* Narakusumo & Riedel, sp. nov., holotype **a** habitus **b** penis.

***Holotype*: Female** (Fig. 6.a) Length 2.95 mm. Color of antennae ferruginous, head and legs dark ferruginous, remainder black. Body subovate; in dorsal aspect with weak constriction between pronotum and elytron; profile dorsally convex. Eyes dorsally approximate. Rostrum in basal 1/3 with median ridge and pair of submedian ridges; with suberect white scales; in apical half dorsally flattened, subglabrous, punctate Pronotum densely punctate with small punctures. Elytra subglabrous; striae marked by rows of minute punctures; intervals subglabrous and with row of even smaller punctures; along base and behind humerus bordered by row of deep punctures. Femora with distinct anteroventral ridge, near middle with denticle. Mesofemur and metafemur dorsally densely squamose with white scales. Metafemur with smooth dorsoposterior edge; subapically without stridulatory patch. Abdominal ventrites 1–2 flat, subglabrous, sparsely punctate, with sparse scales; ventrite 5 flat, punctate, sublaterally with suberect scales. Genitalia (Fig. 5b).

#### Material examined.

Holotype (MZB): MZB0023 (GenBank # MN322592), Indonesia, Maluku, Tanimbar, Selaru Is, Bangruti, 08°07.253'S, 131°02.947'E, 35 m, beaten, 22-IV-2018.

#### Distribution.

Maluku Prov., Tanimbar (Selaru Is). Elevation 35 m.

#### Biology.

On foliage in lowland forest.

#### Etymology.

This epithet is an adjective derived from the species´ type locality, Selaru Island.

#### Notes.

This species is closely related to the undescribed *Trigonopterus* species 436 from Kai Kecil Island, from which it differs by a larger body size and a more densely punctate pronotum and an 8.9% p-distance of its *cox1* sequence.

### Trigonopterus tanimbarensis

Taxon classificationAnimaliaColeopteraCurculionidae

Narakusumo & Riedel sp. nov.

DCBFC61C-E4C8-5948-A8A0-874FF7388376

http://zoobank.org/D47905D5-2D81-4721-90E7-853F8714C52E

#### Diagnostic description.

***Holotype*. Male** (Fig. 6a). Length 3.06 mm. Color of antennae and tarsi ferruginous, remainder black. Body slender subovate; profile dorsally convex; in dorsal aspect and in profile with weak constriction between pronotum and elytron. Rostrum dorsally with median ridge somewhat flattened at level of antennal insertion; with fine submedian ridges; in basal 1/2 with dense silvery scales, in apical 1/2 with suberect setae. Pronotum densely punctate; punctures becoming larger anterolaterad; each puncture containing short seta; with subglabrous midline. Elytra with striae marked by rows of small punctures and fine hairlines; basal margin bordered by transverse row of deeper punctures; intervals subglabrous, with interspersed minute punctures. Femora with anteroventral ridge weakly crenate, ending in apical half with small tooth. Metafemur dorsally with recumbent silvery scales; dorsoposterior edge indistinct, weakly denticulate-crenate, subapically with stridulatory patch. Abdominal ventrites 1–2 concave, coarsely punctate, at middle subglabrous; ventrite 5 with shallow impression, densely punctate, sparsely setose, laterally with sparse scale. Penis (Fig. 6b) with sides subparallel, apex subangulate, setose; transfer apparatus complex; sclerites of endophallus and orifice asymmetrical; apodemes 2.1× as long as body of penis; ductus ejaculatorius with indistinct bulbus. **Intraspecific variation.** Length 2.75–3.25 mm. Female rostrum slender, dorsally with subglabrous median costa, with sublateral rows of punctures, in basal 1/3 with sparse suberect scales. Female abdominal ventrites 1–2 flat. Female abdominal ventrite 5, punctate.

#### Material examined.

***Holotype*** (MZB): MZB0010 (GenBank # MN322598), Indonesia, Maluku, Tanimbar, Yamdena Is, Lorulun, 07°48.788'S, 131°22.443'E to 07°48.137'S, 131°21.873'E, 140 m, beaten, 02-V-2017. ***Paratypes*** (MZB, SMNK): Indonesia, Maluku, Tanimbar: 7 exx, MZB0008 (GenBank # MN322600), MZB0009 (GenBank # MN322599), MZB0011 (GenBank # MN322593), same data as holotype; 4 exx. MZB0035 (GenBank # MN322596), Yamdena Is, Lorulun, Jungle Camp, 07°46.46'S, 131°20.482'E, 110 m, beaten, 19-IV-2018; 53 exx. MZB0028 (GenBank # MN322597) MZB0037 (GenBank # MN322594), Yamdena Is, Lorulun, Jungle Camp, 07°46.46'S, 131°20.482'E, 110 m, beaten, 24-IV-2018; 1 ex., MZB0036 (GenBank # MN322595), Yamdena Is, Lorulun, Jungle Track, 07°47.396'S, 131°20.849'E, 120 m, beaten, 19–20-IV-2018.

#### Distribution.

Maluku Prov., Tanimbar (Yamdena Is). Elevation 110–140 m.

#### Biology.

On foliage in lowland forest.

#### Etymology.

This epithet is an adjective derived from the Tanimbar Archipelago.

#### Notes.

This species appears related to a species from New Guinea (species 959) from which it is differs by 19.9% p-distance of its *cox1* sequence and many morphological characters.

### Trigonopterus triradiatus

Taxon classificationAnimaliaColeopteraCurculionidae

Narakusumo & Riedel sp. nov.

AA421CCF-0FB9-5AA5-9714-9E68D4C0F30D

http://zoobank.org/4FE8402C-4899-4C13-AD16-B4DC50990282

#### Diagnostic description.

**Figure 7.**
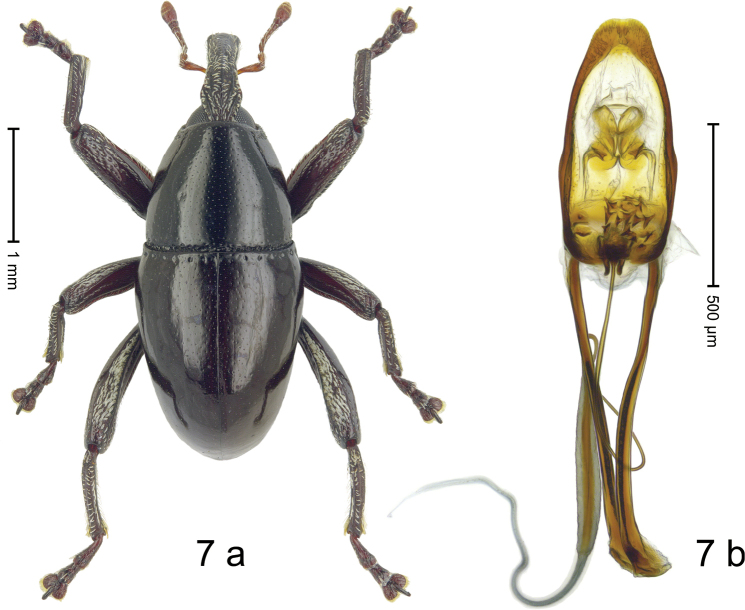
*Trigonopterus triradiatus* Narakusumo & Riedel, sp. nov., holotype **a** habitus **b** penis.

***Holotype*. Male** (Fig. 7a). Length 3.03 mm. Color of antennae ferruginous, legs dark ferruginous, remainder black. Body subovate; in dorsal aspect and in profile with weak constriction between pronotum and elytron. Eyes dorsally approximate. Rostrum in basal 1/2 with median ridge and pair of sublateral ridge; intervening furrows with suberect scales; apical half subglabrous, punctate. Pronotum with disk subglabrous, with minute sculpture; in basal 1/3 laterally with indistinct edge lined with few coarse punctures; laterally anterior margin lined by few white scales, behind eye with row of coarse punctures. Elytra subglabrous, with minute punctures. Femora edentate; with distinct anteroventral ridge. Mesofemur and metafemur dorsally densely squamose with white scales. Metafemur with smooth dorsoposterior edge; subapically without stridulatory patch. Abdominal ventrites 1–2 medially concave, subglabrous, laterally with sparse white scales; ventrite 5 at middle with shallow impression, weakly punctate, with sparse short setae. Penis (Fig. 7b) with sides converging to slightly spatulate apex; transfer apparatus complex; endophallus with triradiate sclerites; apodemes 1.5× as long as body of penis; ductus ejaculatorius with distinct bulbus. **Intraspecific variation.** Length 2.68–3.53 mm. Female rostrum slender, subglabrous, sparsely punctate, in basal 1/4 with indistinct ridges and sparse suberect scale. Female abdominal ventrite 5 flat, subglabrous, with small punctures.

#### Material examined.

***Holotype*** (MZB): MZB0007 (GenBank # MN322604), Indonesia, Maluku, Tanimbar, Yamdena Is, Lorulun, 07°48.788'S, 131°22.443'E to 07°48.137'S, 131°21.873'E, 140 m, beaten, 2-V-2017. ***Paratypes*** (MZB, SMNK): Indonesia, Maluku, Tanimbar: 12 exx, MZB0004 (GenBank # MN322607), MZB0005 (GenBank # MN322606), MZB0006 (GenBank # MN322605) same data as holotype; 3 exx, Yamdena Is, Lorulun, 07°48.788'S, 131°22.443'E to 07°48.137'S, 131°21.873'E, 140 m, beaten, 28–29-V-2017; 7 exx, MZB0029 (GenBank # MN322601) MZB0030 (GenBank # MN322602) Yamdena Is, Lorulun, Jungle Camp, 07°46.46'S, 131°20.482'E, 110 m, beaten, 24-IV-2018; 1 ex, Yamdena Is, Lorulun, Jungle Camp, 07°46.46'S, 131°20.482'E, 110 m, beaten, 19-IV-2018; 1 ex, MZB0021 (GenBank # MN322608) Larat Is, margin of Nature reserve, 07°08.22'S, 131°49.49'E, 40 m, 25-IV-2018; 1 ex, MZB0041 (GenBank # MN322603) Larat Is, Nature reserve, 07°08.747'S, 131°49.092'E, 85 m, beaten, 25–26-IV-2018; 2 exx, MZB0025 (GenBank # MN322609), MZB0112 (GenBank # MN322610), Selaru Is, Bangruti, 08°07.253'S, 131°02.947'E, 40 m, beaten, 22-IV-2018.

#### Distribution.

Maluku Prov., Tanimbar (Yamdena Is, Larat Is, Selaru Is). Elevation: 40–140 m.

#### Biology.

On foliage in lowland forest.

#### Etymology.

This epithet is an adjective based on triradiate sclerites in the endophallus of the species.

#### Note.

This species is closely related to *Trigonopterus* species 60 from Papua New Guinea from which it differs by the structure of the penis and a 17.8% p-distance of its *cox1* sequence.

##### Key to the *Trigonopterus* species of the Tanimbar Archipelago

**Table:** 

1	Metafemur subapically with stridulatory patch. Pronotum densely punctate	**2**
–	Metafemur subapically without stridulatory patch. Pronotum subglabrous, sparsely punctate with small or minute punctures	**3**
2	Elytra black. Basal half of rostrum and forehead with silvery scales not covering the surface. Side of pronotum anteriorly without scales	***T. tanimbarensis* sp. nov.**
–	Elytra ferruginous. Basal half of rostrum and forehead almost covered by white scales. Side of pronotum anteriorly with white scales	***T. porg* sp. nov.**
3	Femora with simple anteroventral ridge; edentate	**4**
–	Femora with ventral denticle	**6**
4	Eyes medially approximate; base of rostrum much wider than forehead between eyes. Apex of male mesotibia only with uncus	**5**
–	Base of rostrum subequal to forehead between eyes. Apex of male mesotibia with enlarged premucro	***T. laratensis* sp. nov.**
5	Body slender. Pronotum sparsely punctate. Elytral striae marked by rows of minute punctures and fine hairlines	***T. atuf* sp. nov.**
–	Body wider. Pronotum subglabrous. Elytra subglabrous, without distinct striae	***T. triradiatus* sp. nov.**
6	Elytral base bordered by row of deeper punctures. Apex of metatibia with supra-uncal denticle	***T. selaruensis* sp. nov.**
–	Elytral base without deeper punctures (except row hidden behind humerus). Apex of metatibia dorsally of uncus rounded	***T. kumbang* sp. nov.**

## Discussion

*Trigonopterus* had hitherto been recorded from Ceram Island (Riedel 2011), Flores of the Lesser Sunda Islands (Riedel et al. 2014), the Aru Islands (Pascoe 1885), Eastern Australia (Riedel and Tänzler 2016), and New Guinea (Riedel 2011, Riedel et al. 2013b). The newly discovered *Trigonopterus* fauna of the Tanimbar Islands fills a gap in the known distribution and is of special interest due to the isolated position and recent geological age of these islands. The islands of Sumba, Alor, Timor, and Wetar should be searched for additional undescribed species in future.

All the discovered species of Tanimbar *Trigonopterus* live on foliage, and no edaphic lineage could be found. This may be due to the relatively dry climatic conditions, which may be putting stress on species that depend on a layer of moist litter. Alternatively, it is possible, that edaphic species are present but have eluded discovery so far; sifting of leaf litter under the right conditions, e.g., after sufficient rainfalls, may bring them to light.

Morphologically the Tanimbar *Trigonopterus* species are very different from each other, a fact supported by the molecular dataset of their *cox1* sequences. Therefore, no closely related species pairs can be recognized, i.e., there is no indication for any autochthonous speciation on the Tanimbar Archipelago. Instead, the *Trigonopterus* fauna has been formed largely by repeated dispersal from neighboring regions, i.e., from Western New Guinea and the Moluccas. The sister species of *T. porg* sp. nov. (13.6% *p*-distance of *cox1*) and *T. selaruensis* sp. nov. (8.9% *p*-distance of *cox1*) were both found on Kai Kecil Island 190 km to the Northeast. *Trigonopterus triradiatus* sp. nov. is related to *Trigonopterus* species 60 from Papua New Guinea (17.8% *p*-distance of *cox1*). *Trigonopterus laratensis* sp. nov. belongs to a clade comprising *T. allotopus* Riedel, *T. pseudallotopus* Riedel, and some undescribed species from New Guinea (15.6% *p*-distance of *cox1*), but has no close relationship to the clade of Australian species of the *T. politus*-group. *Trigonopterus kumbang* sp. nov. belongs to the *T. nasutus*-group and appears most closely allied to *Trigonopterus* species 929 (12.3% *p*-distance of *cox1*) from the D’Entrecasteaux Islands. *Trigonopterus atuf* sp. nov. is closely related to *Trigonopterus* species 773 (15.1% *p*-distance of *cox1*) from Papua New Guinea. *Trigonopterus tanimbarensis* sp. nov. appears related to a species from New Guinea (*Trigonopterus* species 959; 19.9% *p*-distance of *cox1*).

With its close proximity to Australia, stronger ties to the Australian fauna could be expected, but apparently this is not the case. An explanation could be that the Australian species are largely restricted to the Cape York Peninsula and the east coast of Queensland, which is quite distant from the Tanimbar Islands, and that the absence of *Trigonopterus* from the Northern Territory in Australia could be a real gap in the distribution of the genus and not just a sampling artifact, caused by environmental extremes.

All in all, the observed composition of the *Trigonopterus* fauna of the Tanimbar Archipelago is exactly what can be expected from the geological setting and what has been observed in other taxa (How and Kitchener 1997; Beck et al. 2006; Michaux 2010; Andersen et al. 2013): 1) a relatively recent origin that may not have allowed for local speciation and 2) an insular situation not compromised by periods of low sea level. This is quite different in the otherwise similar Aru Islands further east which were part of the continuous Sahul shelf during the Pleistocene. However, no focused fieldwork has ever been carried out on the Aru Islands, from which only *T. oblongus* Pascoe is known to date. Presumably, further collecting on these islands would discover additional species with stronger ties to the Southern Papuan fauna.

The rapid and ongoing anthropogenic activities in Tanimbar, i.e., agriculture and forestry, put pressure on the natural forests of the islands, which are the exclusive habitats of *Trigonopterus* species. The first author found the southern part of Yamdena Island to be extensively logged, and most areas of the eastern coast have been converted to agriculture and settlements. The forests of Larat Island are also severely affected by agriculture, with coconut plantations prevalent inside the wildlife conservation area. Finally, Selaru Island without any protected areas, has suffered worst from logging; its interior has already been turned into grassland and the remaining forests areas are fragmented on the sparse rocky soil that is almost useless for gardening. Such destructions of natural forest areas in Tanimbar threaten not only the endemic *Trigonopterus* species but also the remaining biodiversity of this fascinating archipelago.

## Supplementary Material

XML Treatment for Trigonopterus

XML Treatment for Trigonopterus atuf

XML Treatment for Trigonopterus kumbang

XML Treatment for Trigonopterus laratensis

XML Treatment for Trigonopterus porg

XML Treatment for Trigonopterus selaruensis

XML Treatment for Trigonopterus tanimbarensis

XML Treatment for Trigonopterus triradiatus
